# Lifetime cost-effectiveness of myopia control intervention for the children population

**DOI:** 10.7189/jogh.14.04183

**Published:** 2024-09-20

**Authors:** Ching So, Jinxiao Lian, Sarah Morag McGhee, Rita Wing Man Sum, Andrew Kwok Cheung Lam, Maurice Keng Hung Yap

**Affiliations:** 1 School of Optometry, The Hong Kong Polytechnic University, Hong Kong SAR, China; 2 Public Health Research Group, School of Optometry, The Hong Kong Polytechnic University, Hong Kong SAR, China; 3 Research Centre for SHARP Vision (RCSV), The Hong Kong Polytechnic University, Hong Kong SAR, China; 4 School of Public Health, The University of Hong Kong, Hong Kong SAR, China

## Abstract

**Background:**

Myopia is a common eye condition and projected to affect half of the global population by 2050. Controlling its progression during childhood may prevent associated ocular diseases in later life. Certain interventions retard myopia progression but their long-term costs and consequences are not well understood. We evaluated the cost-effectiveness of myopia control via an optical approach using the Defocus Incorporated Multiple Segments (DIMS) lens over a lifetime.

**Methods:**

We constructed an individual-based, state-transition model to simulate 1) the development and progression of myopia in childhood with and without control and 2) the impact of myopia on the development of four sight-threatening complications in adulthood. We compared strategies of myopia control with 100% uptake vs. no myopia control from the societal perspective to determine whether myopia control is value for money.

**Results:**

With myopia control, the cumulative prevalence of high myopia was relatively reduced by 44.7% (5.9 vs. 10.7%) and severe visual impairment by 19.2% (2.2 vs. 2.7%) compared to no myopia control. The lifetime cost per quality-adjusted life year gained was 26 407 US dollars (USD) and is considered cost-effective compared to the threshold recommended by the World Health Organization (WHO) of one times annual per capita gross domestic product (48 359 USD). Probabilistic sensitivity analysis showed that myopia control had an 87% likelihood of being cost-effective at the WHO threshold.

**Conclusions:**

Myopia control is cost-effective when provided to all eligible children. Further investigation is required to determine if it is cost-effective for the government to subsidise myopia control in order to maximise access.

Myopia is a global problem, affecting 34% of the world population in 2020 and expected to reach 50% by 2050 [1]. What further makes this condition a concern is the increasing prevalence of high myopia, from 5.2 to 9.8% over the same time period, which increases the risk of developing ocular complications beyond the blurred distance vision that can be corrected by glasses, contact lenses or surgery. Each additional dioptre (D) increase in level of myopia has been associated with an increase of 20% in the risk of open-angle glaucoma, 21% in risk of posterior subcapsular cataract, 30% in risk of retinal detachment, and 58% in risk of myopic maculopathy [2]. Many of these conditions can lead to irreversible vision loss [3–6] with consequences for quality of life [7,8], the economic burden of extra health care costs [9], and loss of productivity due to severe visual impairment [10]. It is foreseeable that the rising prevalence of high myopia will intensify the burden on an already overwhelmed secondary ophthalmic service due to the ageing of populations.

Across the lifetime, the school years often feature the fastest progression of myopia. This is a crucial time period for myopia control as the eye grows throughout childhood, levelling off in early adulthood. Several optical and pharmaceutical interventions have been developed to control the progression of myopia [11,12]. These interventions all have upfront costs but potential benefits include avoidance of ocular complications and maintenance of future quality of life. Therefore, whether myopia control in school children represents an efficient use of resources is an important question for policymakers.

Economic evidence on myopia control is very limited with only one cost-effectiveness analysis (CEA) found in a recent systematic review [13]. This CEA used a Markov cohort model to show that, from a societal perspective, introducing a hypothetical programme of screening for myopia using photorefraction, plus control of progression with 0.01% atropine, for children aged 11 years old was a cost-effective strategy compared to usual care with corrective lenses and optometry follow-up [14]. This is the first proof-of-concept of the potential cost-effectiveness of myopia control but it used a simplified structure by modelling the transition from high myopia to related ocular complications only as a broad category without considering each complication, i.e. glaucoma, cataract, myopia maculopathy, and retinal detachment, separately. Since each type of complication has a different pattern of progression, treatment and follow up and a different impact on individual’s quality of life, there is a need for some more detailed cost-effectiveness modelling. Furthermore, this study overlooked the potential costs and benefit of myopia control for those who develop myopia after the one-off screening; almost half of the 90% of children who were not myopic at screening would be missed if myopia prevalence is 50%. The cost-effectiveness of myopia control through optical approaches has also not been assessed. Another recently published study examined myopia prevention and control strategies for schoolchildren in China [15], but only included impacts during childhood from six to 18 years of age and did not consider complications associated with myopia in later life. It also unable to provide comprehensive evidence for cost-effectiveness of myopia control. Agyekum et al. estimated the cost-effectiveness of different myopia control intervention, but again only in the short term [16]. A study by Fricke et al. estimated the lifetime costs of different myopia management options but focused on the economic efficiency without quantifying the health benefits, and provided only partial information on the cost-effectiveness of myopia control [17].

The aim of this study was to comprehensively evaluate whether myopia control is value for money in retarding myopia progression and preventing future visual impairment, and to further understand the long-term impact on consequent health services utilisation, costs and quality of life. This study was not intended to compare different myopia control interventions (i.e. spectacles, contact lenses and pharmaceutical agents) as each has its own eligibility criteria, and regime features. Local conditions such as availability and compliance issues also need to be considered. This CEA used the example of spectacles as a non-invasive intervention and suitable for most children, as a potential population-based approach, to demonstrate the lifelong impact of myopia control. This evaluation is based on the Defocus Incorporated Multiple Segment (DIMS) lenses, a spectacle lens which has been shown to slow down the progression of myopia by 52% [18], with effect sustainable over the long-term; it has no rebound effect after discontinuation [19] and has been widely adopted in Hong Kong (HK).

## METHODS

A cost-effectiveness model was built to simulate the long-term impact on costs and consequences of starting a myopia control intervention in childhood. The provision of DIMS lenses to children aged between six and 11 years old, in Hong Kong, who are eligible for the intervention was assumed (base case strategy) and was compared with no specific myopia control (reference strategy). The base case analysis assumed 100% uptake of intervention to reflect whether myopia control is value for money in the absence of barriers to acceptance, and to evaluate the maximum impact of myopia control. Scenario analyses were conducted to investigate the impact of discount rates, uptake rates and simulating a younger population for myopia control on cost-effectiveness.

### Model structure

An individual-based state-transition model was used which allows us to include heterogeneity in the development of myopia, response to the benefit of myopia control and development of ocular complications later in adulthood. This model could also capture the development of more than one ocular complication across an individual’s lifetime. The model was built in TreeAge Pro Suite 2020 (TreeAge Software, Inc, Williamstown, MA).

**Figure 1:**
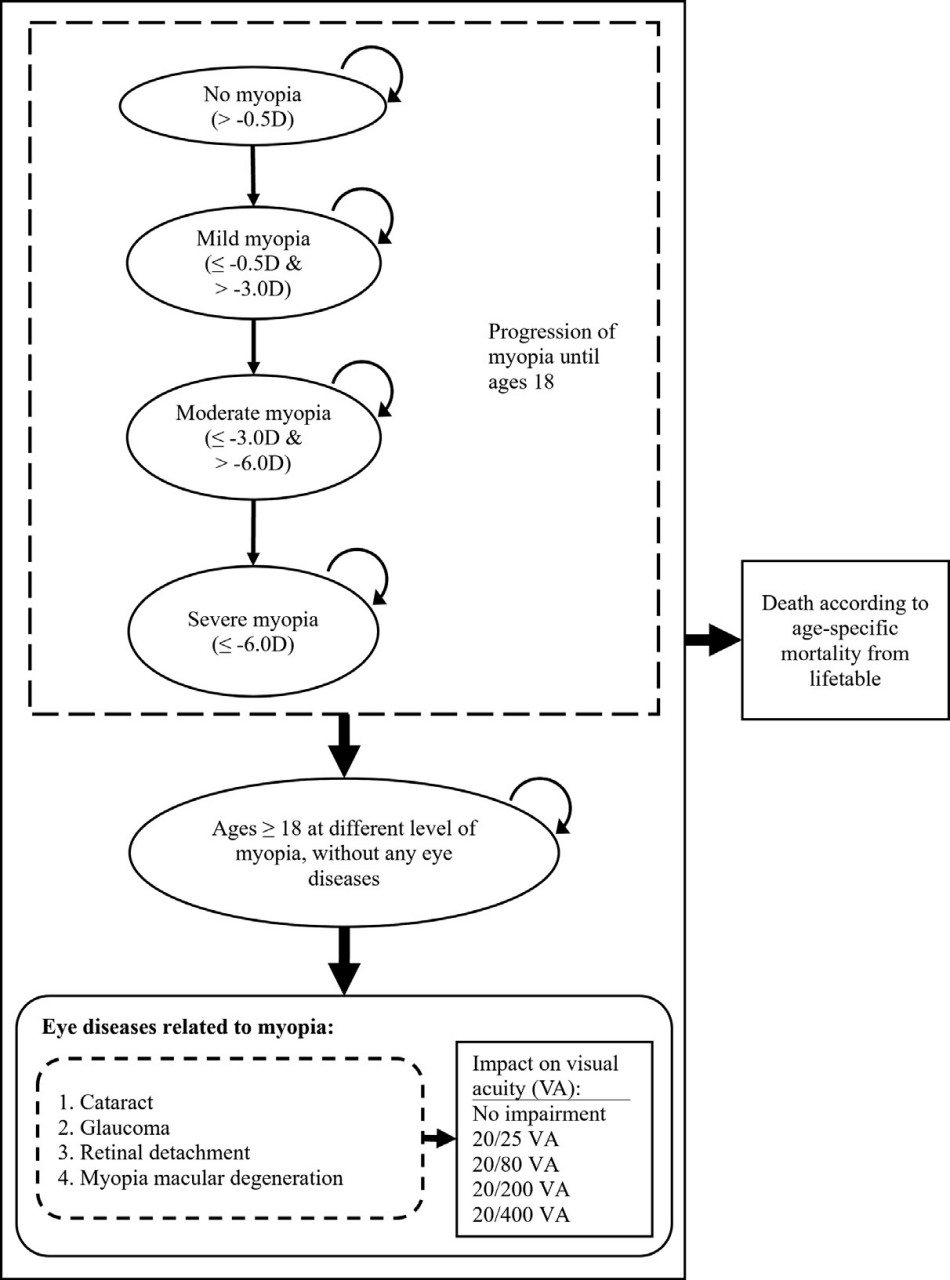
Simulation of natural progression of myopia and related ocular complications. D – dioptre, VA – visual acuity.

The model was based on the natural history of 1) myopia development and progression, including no, mild, moderate, and high myopia states in childhood and 2) the development of four related ocular complications including retinal detachment (RD), myopic macular degeneration (MMD), cataract, and open-angle glaucoma (OAG) during adulthood (**Figure 1**). Transitions between health states were simulated in yearly cycles across the individual’s lifetime.

#### Childhood simulation

The model simulated a hypothetical cohort of 100 000 individuals aged six to 11 years old. All individuals began with a randomly generated profile of different age, sex, and level of myopia in terms of spherical equivalent refraction (SER). The individuals were allocated to one of four health states based on their SER: 1) no myopia,>−0.5 D; 2) mild,≤−0.5 to>−3.0 D; 3) moderate,≤−3.0 to>−6.0 D; and 4) high myopia,≤−6.0 D [20]. Starting from the first cycle, each individual has a onetime chance to initiate the myopia control intervention if the individual is clinically eligible. The individual could have a chance to withdraw after receiving myopia control for the first year but, if they continue, they are assumed to have full compliance until age 18. In each cycle, the development and progression of myopia for each individual is based on annual changes in SER according to the individual’s age, myopia level and whether they received the myopia control intervention, in which case the annual change in SER is reduced. If an individual survives this model cycle, the profile in terms of age, myopia level, and myopia control status is updated for the next model cycle and the above simulation repeats. Given that the level of myopia in most people stabilises by age 18 and the efficacy of existing interventions in younger adults is uncertain [21], the simulation of myopia development and progression stops at age 18 for each individual, with the myopia status carried over to adulthood.

#### Adulthood simulation

Before the age of 50, the four myopia related complications, i.e. RD, MMD, cataract, and OAG are uncommon. Therefore, between the ages of 19 and 49, there is no simulation of complications, only an age-related risk of death. Annual probabilities of developing the four myopia-related eye diseases are applied from age 50 onwards. Individuals who have myopia, particularly high myopia, are subject to higher probabilities of developing the eye diseases and more than one disease is possible across the lifetime. After developing any myopia-related eye disease, the impact on vision is further simulated. Each complication has a different risk of visual impairment, chance of diagnosis, and treatment effectiveness. The progression and decision paths for each eye disease were simulated independently in the model by considering each natural disease history (Figures S1–4 in the **Online Supplementary Document**). Utility decrement values were assigned based on the largest decrement among the disease states that the individual could have. Any individual progressing to severe visual impairment i.e. visual acuity (VA)<20/200, from any eye disease would have no further simulation of other diseases and the utility decrement for severe visual impairment would be assigned to the remaining lifetime. The simulation continued until the individuals reached 100 years old or died.

### Model parameters

A three-level strategy was adopted to identify the best available data sources for parameter values and their ranges. Data from the HK population were used where possible and, if not available, overseas data with adjustment and validation to fit local circumstances were applied. When both were unavailable, estimates based on expert opinion were used. An expert panel, consisting of three senior optometrists practicing myopia control using optical lenses in clinical practice, was set up to review the assumptions and parameter values independently.

#### Myopia development and progression

Census data from HK were used to generate the individual profiles of age and sex of the children in the model. Individuals were then randomly assigned a myopic state (i.e. no, mild, moderate, and high myopia) according to the age-specific prevalence of myopia found among HK primary school children [22]. Adjustment was made based on an assumption that the prevalence in the older age group should not be lower than the previous age group. According to the level of myopia, a random value for SER was generated for each individual based on the possible range for each myopia states, i.e. no myopia (>−0.5 to +3.5 D), mild (>−3.0 to −0.5 D), moderate (>−6.0 to −3.0 D), and high myopia (−12.0 to −6.0 D), based on the distributions which best matched the prevalence data in HK.

The ranges of change in SER by year were derived based on longitudinal studies in HK [23–27] and clinical observation by project panel experts, while the maximum estimate within the range considered all possible variations. The annual change in SER in each model cycle was generated randomly using the Program Evaluation and Review Technique (PERT) distribution [28] with the ranges for myopic children aged 6–7, 8–13, and 14–17 of −1.0 to 0 D, −2.0 to 0 D, and −1.5 to 0 D respectively and −0.5 to 0 D, −1.0 to 0 D, and −0.5 to 0 D respectively for non-myopic children (Figure S5 in the **Online Supplementary Document**). It has the flexibility to modify minimum, maximum, flatness, and skewness of the distribution, and feasible for generating annual SER change which is negatively skewed [23]. Calibration was used to refine the shape parameters defining the PERT distribution until the simulated proportion of myopia cases at age 18 was comparable to the observed prevalence of myopia among young adults in HK [29].

#### Efficacy of DIMS lens on myopia control

The efficacy of the DIMS lens was taken from a two-year randomised trial of DIMS in HK [18]. Individuals who used DIMS lenses were assumed to have a 52% reduction in the change of SER compared to no control in base case, with sensitivity analyses testing the possible range of efficacy from literature. The parameters regarding eligibility and compliance with the use of DIMS lenses were based on clinical practice and observation of expert panel, as there is no such data available in the literature. The proportion of individuals who are clinically eligible for DIMS was assumed to be 95% of those aged six to 15 and who have SER value between −6.0 and −0.5 D. The likelihood of withdrawing in the first year was assumed to be 2% in base case since it is rarely observed based on clinical experience and tested up to 12% in sensitivity, referencing the dropout rate within 12 months in the DIMS study [18]. No adverse effect due to DIMS lenses was included in the model as it is non-invasive and no significant adverse effect has been reported so far [19,30].

#### Transition probabilities among health states

Age and sex-specific mortality rates were based on HK life Tables [31]. No increased risk of mortality for individuals with severe visual impairment was considered in the model since the causal relation between severe visual impairment and death is not well understood. Probabilities of developing the four myopia-related eye diseases were based on overseas studies because HK data were not available and they are described below.

#### Retinal detachment

Age-specific incidence of RD for those aged 50 and above was taken from a retrospective chart review study in Japan in which socioeconomic status and myopia prevalence were similar to HK (**Table 1**) [32]. However, they reported average age-specific incidences, not incidence by level of myopia. Therefore, a population attributable risk approach was applied to decompose the average incidence rates into specific rates for the different levels of myopia. The pooled odds ratios (OR) for developing RD by level of myopia (reference level is no myopia) were obtained from a meta-analysis [33] and multiplied with the incidence rate in the no myopia group to reflect the increased risk of RD with higher levels of myopia. Individuals who developed RD were assumed to have an 80% chance of visiting an ophthalmologist and receiving treatment. This was based on the compliance rate (80.8%) to ophthalmologist referral for sight-threatening diabetic retinopathy from a previous RCT in HK [35]. Those treated successfully for RD were assumed to retain a VA level of 20/25 or 20/80, depending on whether the macula was involved, regardless of the treatment option [42]. All treated individuals would have one ophthalmologist follow-up in every subsequent cycle. The chance of unsuccessful treatment was assumed to be 19% for an individual without high myopia and 34% for those with high myopia [34]. Those with unsuccessful treatment or who were untreated were assumed to have VA<20/200. The simulation flow is shown in Figure S1 in the **Online Supplementary Document**.

**Table 1:** Parameters for effectiveness in the CEA model

Variables	Base value	Range for test		Distribution for probabilistic sensitivity	Source
		**Lower bound**	**Upper bound**		
**Prevalence of myopia**					Choy et al. [22]
6 y old					
*No myopia*	86.7%				
*Mild myopia*	10.5%				
*Moderate myopia*	1.6%				
*High myopia*	1.2%				
7 y old					
*No myopia*	70.0%				
*Mild myopia*	26.7%				
*Moderate myopia*	2.1%				
*High myopia*	1.2%				
8 y old					
*No myopia*	57.3%				
*Mild myopia*	35.6%				
*Moderate myopia*	5.9%				
*High myopia*	1.2%				
9 y old					
*No myopia*	61.9%				
*Mild myopia*	28.7%				
*Moderate myopia*	8.2%				
*High myopia*	1.2%				
10 y old					
*No myopia*	46.4%				
*Mild myopia*	32.1%				
*Moderate myopia*	17.9%				
*High myopia*	3.6%				
11 y old					
*No myopia*	45.3%				
*Mild myopia*	32.9%				
*Moderate myopia*	18.2%				
*High myopia*	3.6%				
					
**Initial SER range (dioptre), distribution**					
No myopia	>−0.5 to +3.5, triangular				Estimate
Mild myopia	>−3.0 to −0.5, uniform				Estimate
Moderate myopia	>−6.0 to −3.0, uniform				Estimate
High myopia	−12.0 to −6.0, triangular				Estimate
					
**Annual change in SER (dioptre), distribution**					
Myopic					
*6–7 y old*	−1.00 to 0, PERT				Estimate
*8–13 y old*	−2.00 to 0, PERT				Estimate
*14–17 y old*	−1.50 to 0, PERT				Estimate
Non-myopic					
*6–7 y old*	−0.50 to 0, PERT				Estimate
*8–13 y old*	−1.00 to 0, PERT				Estimate
*14–17 y old*	−0.50 to 0, PERT				Estimate
					
**Myopia control with DIMS**					
Effectiveness of DIMS	52%	41%	62%	Uniform	Lam et al. [18]
Dropout of DIMS in the first year	2%	-	12%	-	Estimate
Proportion of adverse effects	0%				Lam et al. [19]
					
**Myopia-related eye diseases**					
**Retinal detachment**					
Annual incidence for no myopia					
*50–59 y old*	0.017%				Haga et al. [32]
*60–69 y old*	0.016%				Haga et al. [32]
*70–79 y old*	0.008%				Haga et al. [32]
*80+ years old*	0.004%				Haga et al. [32]
OR of RD					
*Mild myopia*	3.15	1.92	5.17	Log normal	Haarman et al. [33]
*Moderate myopia*	8.74	7.28	10.50	Log normal	Haarman et al. [33]
*High myopia*	12.62	6.65	23.94	Log normal	Haarman et al. [33]
Probability of postoperative VA<20/200					
*Non-high myopia*	19%				Salicone et al. [34]
*High myopia*	34%				Salicone et al. [34]
Annual probability to treatment	80%				Lian et al. [35]
					
**Myopic macular degeneration**					
Annual transition probabilities					
*No myopia*	0%				
*Mild myopia*	0.15%	0.08%	0.22%	Beta	Wong et al. [36]
*Moderate myopia*	0.32%	0.13%	0.49%	Beta	Wong et al. [36]
*High myopia*	0.35%	0.13%	0.57%	Beta	Wong et al. [36]
Annual progression to more severe state					
*Mild/moderate myopia*	1.81%	0.92%	2.77%	Beta	Wong et al. [36]
*High myopia*	4.39%	2.98%	5.91%	Beta	Wong et al. [36]
Annual probability of progress to VA<20/200					
*Not treated*	25.5%				Yoshida et al. [37]
*Treated*	13.7%				Yoshida et al. [37], Ikuno et al. [38]
Annual probability to treatment	80%				Lian et al. [35]
					
**Cataract**					
Annual incidence (no myopia)					
*Nuclear cataract*	1.96%				Kanthan et al. [39]
*Cortical cataract*	1.66%				Kanthan et al. [39]
*Posterior subcapsular cataract*	0.44%				Kanthan et al. [39]
OR of cataract					
Nuclear cataract					
*Mild myopia*	1.79	1.08	2.97	Log normal	Haarman et al. [33]
*Moderate myopia*	2.39	1.03	5.55	Log normal	Haarman et al. [33]
*High myopia*	2.87	1.43	5.73	Log normal	Haarman et al. [33]
Cortical cataract					
*Mild/moderate/high myopia*	1.00				Haarman et al. [33]
Posterior subcapsular cataract					
*Mild myopia*	1.56	1.32	1.84	Log normal	Haarman et al. [33]
*Moderate myopia*	2.55	1.99	3.28	Log normal	Haarman et al. [33]
*High myopia*	4.55	2.67	7.75	Log normal	Haarman et al. [33]
Annual probability to treatment	10%				Estimate
					
**Open angle glaucoma**					
Annual incidence					
*50–59 y old*	0.030%				Hernández et al. [40]
*60–69 y old*	0.080%				Hernández et al. [40]
*70–79 y old*	0.181%				Hernández et al. [40]
*80+ years old*	0.141%				Hernández et al. [40]
OR of OAG					
*Mild myopia*	1.59	1.33	1.91	Log normal	Haarman et al. [33]
*Moderate/high myopia*	2.92	1.89	4.52	Log normal	Haarman et al. [33]
Annual progression to more severe state					
Untreated					
*Mild to moderate*	25.00%				Hernández et al. [40]
*Moderate to severe*	11.00%				Hernández et al. [40]
*Severe to visual impaired*	10.00%				Hernández et al. [40]
Treated (RR for treated to non-treated)	0.65				Hernández et al. [40]
Annual probability to treatment					
*Mild glaucoma*	50%				Estimate
*Moderate and severe glaucoma*	80%				Lian et al. [35]
					
**Mortality rates by age**	HK lifetable				HK Census [31]
					
**Utility decrements**					
Myopia					
*Mild myopia*	0.060	0.040	0.070	Uniform	Li et al. [41]
*Moderate myopia*	0.070	0.060	0.080	Uniform	Li et al. [41]
*High myopia*	0.080	0.070	0.100	Uniform	Li et al. [41]
Retinal Detachment					
*Treated*	0.260	0.100	0.300	Uniform	Yannuzzi et al. [42]
Myopic Macular Degeneration					
*Category 4*	0.170	0.100	0.230	Uniform	Brown et al. [43]
Cataract					
*Not treated*	0.100	0.000	0.170	Uniform	Brown et al. [43]
Open angle glaucoma					
*Mild myopia*	0.199	0.118	0.253	Uniform	Hernández et al. [40]
*Moderate myopia*	0.253	0.199	0.287	Uniform	Hernández et al. [40]
*High myopia*	0.287	0.253	0.358	Uniform	Hernández et al. [40]
Severe visual impairment (VA<20/200)	0.430	0.310	0.620	Uniform	Brown et al. [43]

CEA – cost-effectiveness analysis, DIMS – Defocus Incorporated Multiple Segments, OAG – open angle glaucoma, OR – odds ratio, PERT – Program Evaluation and Review Technique, RD – retinal detachment, RR – risk ratio, SER – spherical equivalent refraction, VA – visual acuity

#### Myopic macular degeneration

The disease stages of MMD were based on the International Classification from META-analysis for Pathologic Myopia [44], which were: no myopic retinal degenerative lesion (C0); tessellated fundus only (C1); diffuse chorioretinal atrophy (C2); patchy chorioretinal atrophy (C3); and macular atrophy (C4) (Figure S2 in the **Online Supplementary Document**). Category C2 and above were clinically classified as MMD. The six-year myopia-specific incidence and rate of progression of MMD from a Singapore cohort study were converted to annual transition probabilities and applied (**Table 1**) [36]. Starting at age 50, individuals would have a probability of developing MMD every cycle, depending on the level of myopia. We assumed all new incidents were C2 and, once developed, the annual probability of progression to more severe states was applied. Those who progressed to C4 were assumed to have VA 20/40 which was irreversible. Each individual would have an 80% chance of visiting an ophthalmologist and receiving treatment in each model cycle. Anti-vascular endothelial growth factor (anti-VEGF) therapy is the first-line treatment option for myopic choroidal neovascularization (CNV) and was assumed to be used. An untreated individual would have a 25.5% annual probability of progressing to severe visual impairment, based on the published 94.7% probability of having VA<20/200 10 years after the onset of CNV [37]. Treated MMD was assumed to reduce the probability of progression to severe visual impairment by 50%, based on the proportion of CNV patients with improved best-corrected VA after treatment using anti-VEGF in the MYRROR study [38].

#### Cataract

The three cataract subtypes: nuclear cataract (NC), cortical cataract (CC), and posterior subcapsular cataract (PSC), were considered in this CEA model (Figure S3 in the **Online Supplementary Document**). The 10-year incidence rate of the three cataract subtypes for emmetropia from the Blue Mountains Eye Study were applied to the individuals who have no myopia [39]. The pooled ORs from the meta-analysis were used to reflect the risk of cataract associated with myopia [33]. In this model, the individuals could develop one or more cataract subtypes and it would become noticeable at age 60 onwards. The individuals VA was assumed to reduce to 20/25 when untreated, but they would have a 10% chance each year to visit an ophthalmologist and receive treatment. This was based on an expert opinion that 90% would be treated in 20 years. After treatment, the impact on VA would cease. Although there would be an extra risk of developing RD post cataract surgery [45], it was not simulated in this model to avoid double counting with the overall incidence of RD.

#### Open angle glaucoma

Age-specific incidences and transition probabilities of OAG (mild, moderate, severe glaucoma, and visually impaired) states were taken from an economic evaluation of OAG screening [40]. However, the population attributable risk approach was not applied since the available incidence rates were averages across different populations and the prevalence of myopia was unknown. The pooled ORs of developing OAG from a meta-analysis [33] were used to estimate the incidence rate of OAG by myopia states. Annual probabilities of developing OAG were applied when the individual reached age 50. It was assumed that newly developed glaucoma was at a mild level and may progress to more severe states. Those who developed mild OAG would have a 50% chance of visiting an ophthalmologist and receiving treatment in any year while those who developed a moderate or severe level would have an 80% chance. A risk ratio of 0.65 was applied to the probability of progression if the individual was treated. The simulation flow is shown in Figure S4 in the **Online Supplementary Document**.

#### Health utility

A health utility value of one was applied to individuals with no myopia, indicating perfect health with no myopia or eye diseases. A utility decrement, based on published data, was applied to the different myopia states and specific eye diseases (**Table 1**) [40–43,46]. Depending on which state the individual progressed to in that cycle, the corresponding decrement was applied. Only the largest decrement was applied when multiple eye diseases developed. Quality-adjusted life-years (QALYs) were calculated by multiplying the utility value with the time the individual stayed in the corresponding health state.

### Costing parameters

All the costs were derived from local sources in 2019 Hong Kong dollars (HKD) and converted to US dollars (USD) (7.8 HKD ≈ 1 USD) [47]. The costs were estimated from the societal perspective, which included 1) direct cost of optical correction due to myopia, 2) direct cost of myopia control using DIMS lenses, 3) direct cost of follow-up and treatment for myopia-related eye diseases, 4) productivity lost due to severe visual impairment, 5) patient’s time and 6) patient’s travel cost spent due to the myopia control intervention, ophthalmologist follow-up and treatment, and 7) cost of caregiver for those with severe visual impairment (**Table 2**).

**Table 2:** Parameters for costs in the CEA model from the societal perspective

Variables	Base value	Range for test		Distribution for probabilistic sensitivity	Source
		**Lower bound**	**Upper bound**		
**Cost for optical correction per year, mean (USD)**					
Childhood (glasses)					
*Mild myopia*	193	135	251	Uniform	Costing survey (unpublished)
*Moderate/high myopia*	254	193	330	Uniform	Costing survey (unpublished)
Adult (glasses, contact lenses, medical due to contact lenses)					
*Mild myopia*	190	133	247	Uniform	Costing survey (unpublished)
*Moderate myopia*	380	266	417	Uniform	Costing survey (unpublished)
*High myopia*	417	380	542	Uniform	Costing survey (unpublished)
					
**Cost of myopia control intervention (DIMS), USD**					
Cost of a clinic visit for a comprehensive eye examination	83				Estimate (market price)
Cost of a follow-up visit for an eye checking and monitoring	58				Estimate (market price)
First package cost	510	357	663	Uniform	Estimate (market price)
Second package cost	444	311	577	Uniform	Estimate (market price)
Spectacle frame	96	64	128	Uniform	Estimate (market price)
Proportion of spectacle replacement	95%				Estimate
Travel cost due to optometric visit (1 way), USD	0.4				Costing survey (unpublished)
Travel time due to optometric visit (1 way), mins	3.3				Costing survey (unpublished)
Number of accompanying persons	1.1				Costing survey (unpublished)
**Cost for myopia-related eye diseases**					
Retinal Detachment					
*Cost of surgery, USD*	8462				Hospital Authority [48]
*Number of pre- and post-operative follow-ups*	6				Wong et al. [49]
*Productivity loss*					
*Cost per bed day, USD*	654				HKSAR Gazette [50]
*Recovery period*	4 weeks				Estimate
Myopic macular degeneration					
*Cost of anti-VEGF*					
*Number of injections*	3				Ikuno et al. [38]
*Cost per injection, USD*	987				Estimate (market price)
Cataract					
*Cost of cataract extraction, USD*	1936				HKSAR Gazette [50]
*Number of pre- and post-operative follow-ups*	6				Wong et al. [49]
*Productivity loss*					
*Recovery period*	4 weeks				Estimate
Open angle glaucoma					
*Treatment of OAG, USD*					
*Cost of eye drop (yearly)*	462				Estimate (market price)
*Cost of laser trabeculoplasty*	3192				Hospital Authority [48]
*Cost of surgery*	8462				Hospital Authority [48]
**Patient time cost**					
Time cost for specialist clinic, USD	13				Lian et al. [35]
Time cost for travel to specialist clinic (2-way), USD	13				Lian et al. [35]
**Patient travel cost**					
Travel to specialist clinic (2-way), USD	1				Lian et al. [35]
Total patient time and travel cost per specialist visit, USD	27				Lian et al. [35]
Proportion of persons with seeing difficult had a person to take care of their day-to-day living	39%				Census and Statistics Department [51]
Monthly salary for caregiver, USD	594				Market price
Cost of caregiver for blind individual per year, USD	2778				Calculated
Productivity loss due to blindness per year, USD	28 615				Calculated

CEA – cost-effectiveness analysis, DIMS – Defocus Incorporated Multiple Segments, OAG – open angle glaucoma, USD – US dollar, VEGF – vascular endothelial growth factor

The cost of optical correction was based on empirical data about the costs associated with myopia in the past 12 months collected in a pilot costing survey (unpublished). The cost of the DIMS intervention was estimated using an ingredient approach based on the actual market price, including a prior optometrist visit for a comprehensive eye examination (83 USD), a package cost (spectacle lenses and one follow-up visit at 6-month) of 510 USD and spectacle frames (96 USD). After initiating the intervention, the individual would have a routine visit to an optometrist (58 USD) every six months for checking and monitoring. We assumed 95% of the individuals would require spectacle replacement every 18 months and the same items were costed except with a lower package cost (444 USD).

The cost of treatment for myopia-related eye diseases was based on the corresponding treatment options, referring to the cost-recovery value from the HK Government Gazette and Hospital Authority charges for private services [48,50]. For RD, the mid-value (8462 USD) of the available surgery types was applied and required one hospital bed day (654 USD). The patient was assumed to have one preoperative ophthalmologist visit (164 USD) and, postoperatively, five ophthalmologist follow-ups in the treatment year and one ophthalmologist follow-up in every subsequent year, as found in a published study on follow-up for cataract [49]. For MMD, a total of three anti-vascular endothelial growth factor (anti-VEGF) injections, i.e. the first-line treatment option for myopic choroidal neovascularization, was assumed [38]. The cost was 987 USD per injection plus an ophthalmic procedure cost of 93 USD and one ophthalmologist follow-up in every subsequent year after treatment [50]. The cost of cataract extraction included the cost of surgery (1936 USD) and a day ophthalmic procedure [50]. There would be one preoperative and five postoperative ophthalmologist visits in the treatment year but no further follow-up would be required [49]. For treatment of OAG, we assumed the patient would start with glaucoma medication (eye drops, 38 USD per bottle per month) and there would be a onetime chance of 10 and 5% in the second year that the patient required further treatment with trabeculoplasty (3192 USD) and trabeculectomy respectively (8462 USD) [48], based on the Glaucoma Laser Trial and the Follow-up Study [52] and current clinical practice in HK. The latter two conditions would also have the cost of one ophthalmic procedure, one preoperative, and five postoperative ophthalmologist follow-ups.

The median monthly salary (2385 USD) of the HK population was used to estimate the cost of productivity loss [53]. We assumed those who received RD surgery, cataract extraction, and trabeculoplasty or trabeculectomy for glaucoma would have four weeks of productivity lost due to recovery time. Annual productivity loss due to severe visual impairment was calculated by multiplying the median monthly salary by 12 and applying this to the ages up to 65. The time cost for adults accompanying children for myopia control intervention was based on the travel and clinic visit time collected from the pilot costing survey (unpublished), and multiplied by the median salary and transportation expenditures for attendance at health services were derived from the same survey. The time cost for ophthalmologist follow-up and treatment was estimated in a similar fashion based on data from a previous RCT [35]. For caregiver cost, we assumed that 39% of those with severe visual impairment would require caregiver service [51]. The salary of domestic helper (594 USD per month) in HK was applied as staffing costs for providing care.

### Cost-effectiveness analysis

Lifetime costs and benefits were computed and compared. Incremental cost-effectiveness ratios (ICERs) were calculated by dividing the difference in cost by the difference in QALYs. The ICERs were compared with the willingness to pay (WTP) threshold for a QALY. All future costs and QALYs were discounted at 3.5% per year as recommended by the National Institute for Health and Care Excellence (NICE) guideline [54]. Discounting takes into account the impact of when the costs are incurred and outcomes achieved, and adjust them to their present values. Since there is ongoing debate on whether differential discounting rates should be used and evidence is lacking for a positive time preference of parents to their children’s health and quality of life in the future, three scenario analyses were explored: 1) no discount and 5% discount on QALYs, 2) no discount on both costs and QALYs, and 3) 5 and 10% discount on costs. The impact of uptake rate was tested by changing to 50 and 85% to reflect the overall and maximum compliance for free spectacles in a meta-analysis [55]. The impact of simulating a younger children population age six to eight years who may initiate myopia control at a younger age was explored in a scenario. To determine the cost-effectiveness, we referred to the threshold recommended by the World Health Organization (WHO) that a QALY gained for under one times the per capita gross domestic product (GDP) (48 359 USD in Hong Kong, 2019) is very cost-effective [56,57].

One-way sensitivity analyses were conducted to identify the major drivers and assess their impact on cost-effectiveness. This was done by changing the value of the tested parameters one at a time to the lower and upper bounds (**Table 1**, **Table 2**). The test parameters included cost and efficacy of DIMS, cost of refractive correction for each level of myopia, ORs of developing each ocular complication by level of myopia, and utility decrement for each health state. The results are displayed in Tornado Charts. Probabilistic sensitivity analysis was conducted to assess the robustness of the results in considering the uncertainties around the parameters. Random values were drawn from the corresponding distributions of the selected parameters and repeating 1000 times for 1000 individuals to obtain the distribution of ICERs. The included parameters were the same as those in the one-way sensitivity analysis. The results are displayed as a cost-effectiveness acceptability curve.

## RESULTS

The simulation results showed that the cumulative proportion for any myopia by the age of 18 was 77.5% both with and without myopia control. This was because the myopia intervention would not affect the incidence (new cases) of myopia. The proportion of moderate myopia was 20.3% under the myopia control strategy and 32.8% for no myopia control, a 38.1% relative reduction while the proportion of high myopia was 5.9 and 10.7% respectively, a 44.7% relative reduction with myopia control (**Table 3**). The relative reduction in the proportion of individuals who developed RD, MMD, cataract, OAG, and severe visual impairment over their lifetime in the myopia control strategy was 19.5, 15.6, 1.3, 10.2, and 19.2% respectively, compared to no myopia control.

**Table 3:** Simulated prevalence of myopia and eye diseases developed over lifetime with myopia control using DIMS and without control

Variables	Without myopia control (0% uptake)	With myopia control (100% uptake)
**Prevalence of myopia**		
No myopia	22.5	22.5
Any myopia	77.5	77.5
*Mild myopia*	34.0	51.3
*Moderate Myopia*	32.8	20.3
*High Myopia*	10.7	5.9
**Prevalence of eye diseases**		
Retinal detachment	2.2	1.8
Any myopic macular degeneration	6.5	5.5
Any cataract	72.6	71.6
Any glaucoma	7.0	6.3
Severe visual impairment (VA<20/200)	2.7	2.2

DIMS – Defocus Incorporated Multiple Segments, VA – visual acuity

In term of cost-effectiveness, when both costs and QALYs were discounted at 3.5% per year (base case), the incremental cost for myopia control vs. no control was 1293 USD (7357 vs. 6064) per individual across their lifetime which, with the incremental QALY gained of 0.05 (25.89 vs. 25.84), resulted in an ICER of 26 407 USD per QALY gained with myopia control (**Table 4**). The breakdown of cost showed that myopia control intervention would cost 2043 USD per individual over lifetime, but would save 663 USD for optical correction, and 86 USD for costs associated with medical services and vision loss compared to no control.

**Table 4:** Long-term costs and consequences of myopia control using DIMS and no myopia control from the societal perspective (base case)

Variables	Without myopia control (0% uptake)	With myopia control (100% uptake)
**Cost**		
Total costs* (USD)	6064	7357
*Myopia control*	0	2043
*Optical correction*	5433	4770
*Ocular complication*	338	318
*Visual impairment*	292	227
**Incremental cost (USD)**		1293
**Effectiveness**		
Total QALYs*	25.84	25.89
**QALYs gained**		0.05
**Incremental cost per QALYs gained† (USD)**		26407

DIMS – Defocus Incorporated Multiple Segments, QALY – quality-adjusted life year, USD – US dollar

*Average value per individual across lifetime.

†May not exactly equal to the costs divided by QALYs, due to rounding of the decimals.

In the scenario analysis, with a discount rate of 3.5% on costs only but varied the discount rate on QALYs to 0 and 5%, the incremental QALYs gained were 0.16 and 0.03, respectively, resulting in ICERs of 7932 USD and 38 282 USD per QALY gained with myopia control, respectively (Table S1 in **Online Supplementary Document**). With no discounting on both costs and QALYs, myopia control was cost-saving compared with no myopia control. Increasing the discount rate for costs from 3.5% in base case to 5 and 10%, while remained at 3.5% for QALYs, reduced the total lifetime cost per individual but resulted in similar incremental costs and ICERs to the base case results. Reducing the 100% uptake rate of myopia control to 50 and 85% reduced both the incremental cost and QALY gained, but also resulted in lower ICERs of 22 073 USD and 24 488 USD, respectively, compared to base case. When simulated a younger age group of six to eight years old, there was a slight increase in the proportion of preventions of high myopia and ocular complications. This resulted in incremental costs of 1332 USD and QALY gained of 0.05, yielding a lower ICER of 25 031 USD. All ICERs from the base case and scenario analyses were below the WHO threshold of 48 359 USD and were considered cost-effective.

**Figure 2:**
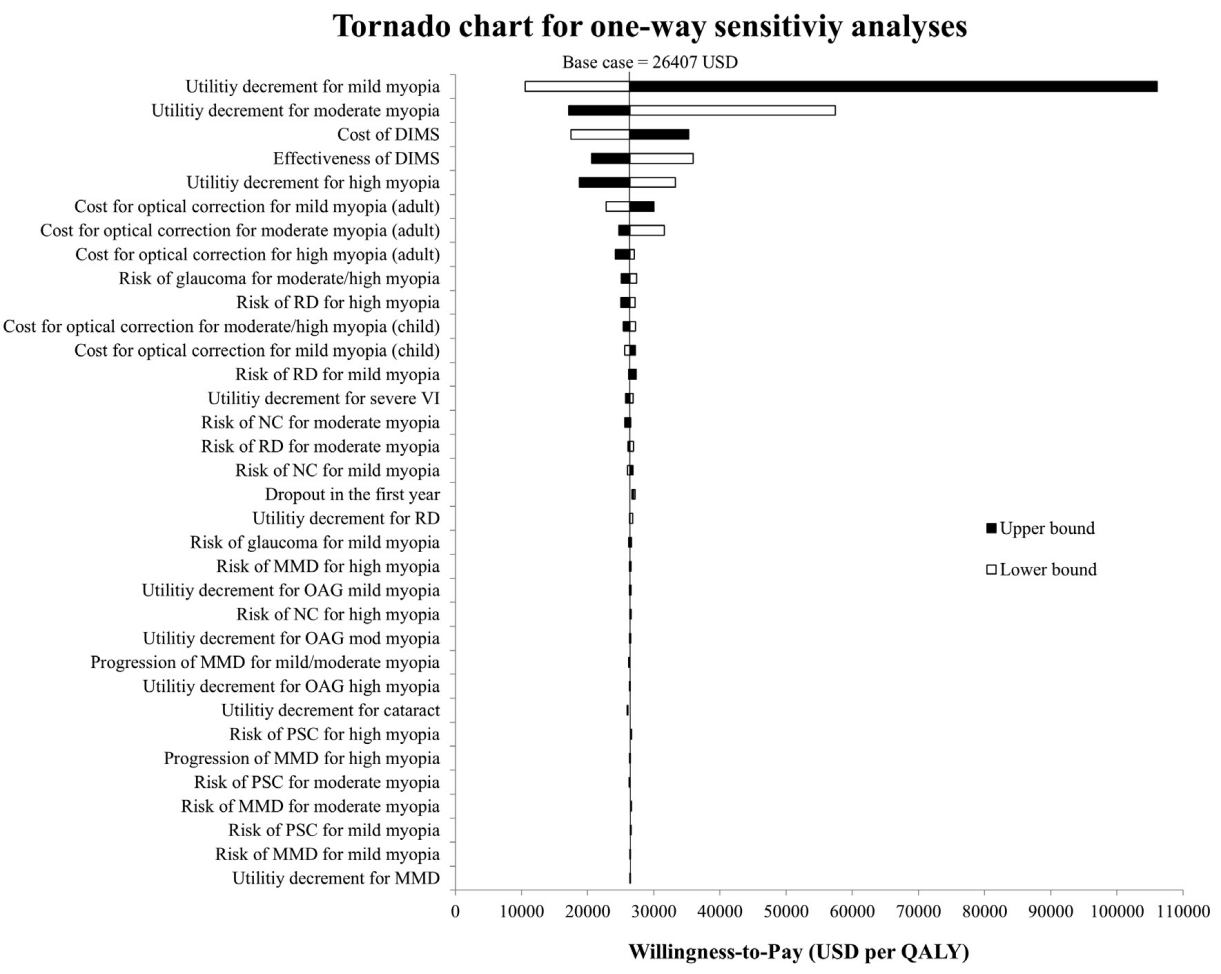
Tornado chart for one-way sensitivity analysis of myopia control using DIMS compared with no myopia control. Black bars represent the ICERs when parameter value changed to the upper bound, white bars represent the ICERs when parameter value changed to the lower bound. DIMS – Defocus Incorporated Multiple Segments, MMD – myopic macular degeneration, NC – nuclear cataract, OAG – open angle glaucoma, PSC – posterior subcapsular cataract, QALY – quality-adjusted life year, RD – retinal detachment, VI – visual impairment.

**Figure 3:**
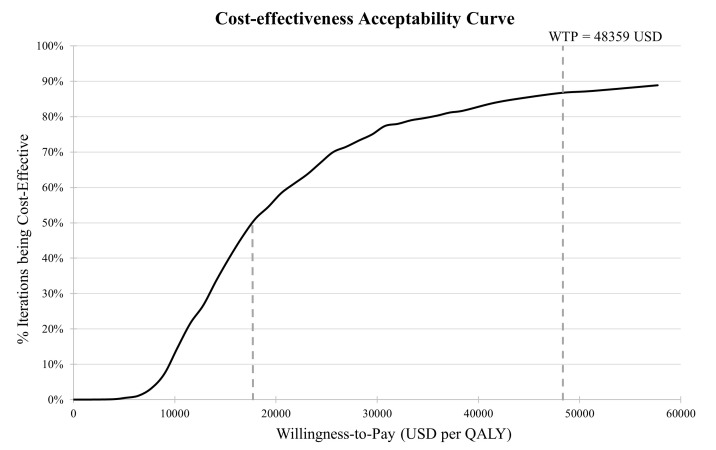
Cost-effectiveness acceptability curve of myopia control using DIMS compared with no myopia control. DIMS – Defocus Incorporated Multiple Segments, QALY – quality-adjusted life year, WTP – willingness-to-pay.

In the one-way sensitivity analysis, the ICERs obtained by varying the cost and effectiveness parameters and the deviation from the base case result were ranked to show the principal drivers of the results. The five parameters with the greatest impact on the ICER were utility decrements for mild, moderate and high levels of myopia, cost of DIMS and effectiveness of DIMS. All ICERs across the test range of parameters’ values were within the WHO threshold (**Figure 2**), except the test on two parameters, i.e. utility decrement on mild myopia and on moderate myopia. The ICER exceeded 48 359 USD per QALY threshold when the utility decrement for mild myopia was tested at the upper bound or for moderate myopia at the lower bound. In the probabilistic sensitivity analysis, possible values of ICER taking into account parameter uncertainties were generated and graphed as an acceptability curve to indicate the proportion at which myopia control is cost-effective at different WTP thresholds. The results showed that, at the WTP for a QALY of 18 000 USD or above, myopia control using DIMS had over 50% probability of being cost-effective, compared to no myopia control (**Figure 3**). When the WTP for a QALY was at the WHO threshold of 48 359 USD, myopia control using DIMS reached 87% probability of being cost-effective.

## DISCUSSION

This is the first cost-effectiveness analysis based on a detailed natural disease history simulation to evaluate the cost, effectiveness, and cost-effectiveness of myopia control using DIMS as an example compared to no myopia control.

Model validation showed that the simulation results mapped well to the observed results in the literature, with the cumulative proportion of myopia in the no myopia control strategy comparable to the observed prevalence of myopia of 9.9% and visual impairment of 2.7% in HK [29,58]. This indicated that our model reasonably reflected the natural disease development and progression of myopia.

The model results showed that myopia control achieved a relative reduction of 38.1 and 44.7% in moderate and high myopia respectively, compared with no control. This was a conservative estimate based on the provision of myopia control intervention to a cohort of children aged 6–11 with prevalence of myopia equivalent to the current child population in HK, some of whom started with a moderate level of myopia, e.g. around 18% of those aged 10 and 11 had moderate myopia. These individuals can only benefit from the intervention for a limited period as their SER was already close to or required only a short time to reach high myopia levels. The intervention would be expected to be of greater benefit if it was offered to all children soon after the diagnosis of mild myopia.

In the base case analysis, myopia control with DIMS was shown to cost 26 407 USD per QALY gained compared with no myopia control. This was considered to be very cost-effective from the societal perspective with an ICER well below one GDP per capita (48 359 USD) in HK, even if all future costs and QALY gains were discounted at 3.5% annually. When the future QALYs and cost discounted, only 10% of lifetime costs were attributed to ocular complications and vision impairment, resulting in relatively small cost saving compared to the cost of the intervention. However, myopia control was cost-effective when taken into account together with the health benefit, which was primarily the maintenance of quality of life by preventing high myopia, associated complication and vision loss.

With no discount on the QALYs but a 3.5% discount on costs, myopia control could result in an extra 0.16 QALYs per individual across their lifetime, lowering the ICER to 7932 USD per QALY gained. Choosing the discount rate that applies to costs and benefits is especially important for preventative programmes, where costs are incurred in the present but benefits are in the future. Furthermore, the costs and benefits of myopia control fall on different individuals, i.e. costs to parents but benefits to children, and there is little evidence in the literature that parents have a positive time preference for their children’s future health or quality of life. Therefore, the ICER based on undiscounted QALYs may more fairly reflect the cost-effectiveness of myopia control vs. no control.

In the scenario with future costs not discounted to their present value, myopia control was cost saving across the lifetime from a societal perspective. Although there were upfront costs associated with myopia control during childhood, there are also future savings in the cost of refractive correction, health care utilisation for ocular complications, productivity loss due to severe vision impairment and patients’ time cost due to treatment and specialist follow-up for ocular complications. The reduction in uptake of intervention did not alter the conclusion that myopia control was cost-effective, although it implied a smaller benefit at a smaller cost. Earlier initiation of myopia control yielded greater benefits at a higher cost but had a limited impact on ICER. More strategically targeting the most needed group may be necessary for optimise its use.

One-way sensitivity analysis in the base case showed that only when the utility decrement applied to mild myopia was at the upper bound and for moderate myopia was at the lower bound, the ICER could exceed the threshold of one GDP. Considering that the benefits of myopia control accrue in the prevention of ocular complications and visual impairment later in life, QALYs gained in the future were discounted over many years, making the overall cost-effectiveness very dependent on the differences in QALYs accumulated in the different myopia states. Small differences between them in utility scores could result in significant changes in the total QALYs gained. Further probabilistic sensitivity analysis showed that, when combining the above and other uncertainties together, myopia control was over 50% likely to be cost-effective at a WTP value of US$18 000 or above for a QALY gained and had almost 90% chance of being cost-effective at the WHO threshold of one-time annual per capita GDP. We therefore conclude that myopia control would still be cost-effective even when considering the uncertainty in utility decrements for mild and moderate myopia.

Our model structure is based on natural disease progression and therefore is externally generalisable in terms of two aspects: a) for other places planning to evaluate the cost-effectiveness for local populations, it can be achieved by replacing the unit price of relevant cost items and local myopia prevalence and progression rate, and b) to evaluate other interventions by changing the associated assumptions and parameters specific to those interventions. The parameters relating to the development and progression of the four ocular complications for different levels of myopia and their impact on vision were based on international studies and meta-analyses, which has good generalisability as well. The findings from this study are generally applicable to other populations facing the high prevalence of myopia, but differences in health care system need to be considered.

Direct comparisons between different interventions are not included due to the different eligibility criteria and features across different types from pharmaceutical, spectacles and contact lenses interventions. Such comparisons would require simulating a children group eligible for all different types of interventions, which could narrow the targeted population. This study established the health economic evidence that myopia control is value for money. Individuals can choose the most suitable intervention, or combination of interventions [11], based on their eligibility and specific needs, under clinical guidance.

A strategic plan for myopia control is undoubtedly needed to reduce the disease and economic burden of myopia-related complications and vision loss. With inadequate knowledge of the cost-effectiveness of myopia control intervention, policymakers may be inclined to prioritise health care resources for treatment and underutilise prevention strategies. Evidence from New Zealand suggested that myopia screening plus atropine 0.01% for progression control was cost-effective [14]. However, their simplified model could not capture all variations in costs and consequences resulting from the development of different myopia-related complications. Furthermore, the cost-effectiveness of other optical methods for myopia control has not yet been evaluated in the literature. The existing evidence may not be sufficient to draw conclusions about the cost-effectiveness of myopia control. The present study used an individual model simulation based on carefully constructed disease progression with parameter values provided by the best available sources in the literature. Our study fills knowledge gaps in cost-effectiveness of optical myopia control intervention and future costs and benefits of control. The model will be continuously updated to include any future myopia control intervention by inputting relevant parameters when effectiveness information becomes available and the intervention is accepted for clinical use.

Having demonstrated that myopia control is a good use of resources from a societal perspective, the next health economics question to address is how to make myopia control more equitably accessible. The high level of, and user responsibility for, all costs associated with the intervention may prevent economically and socially disadvantaged groups from accessing it. Future research should investigate whether subsidising myopia control for specific populations is cost-effective from a government perspective, but designing such a programme requires gathering reliable real-world estimates of the various components of modern health care system. Additional strategies to support wider use of myopia control, for example, educational programme to increase parental awareness and uptake of myopia control could be developed and evaluated. The data will be useful for policymakers to determine the key area for improvement and carry out upcoming public health schemes for myopia prevention and control for the young population.

There were some limitations in this study. The information on the utility decrement for different levels of myopia was based on the adult myopic population in mainland China [41], since there is no locally derived value for HK. These values may lead to inaccurate estimates of the ICER given that the largest driver of QALYs gained came from the utility decrement for mild and moderate myopia. However, there is evidence that vision-related quality of life does not differ significantly between different populations and ethnicities [59,60]. Our probabilistic sensitivity analysis also suggested that even accounting for these uncertainties, the resulting ICER is still likely to be cost-effective. Furthermore, incidence rates that were both age- and myopia-specific for MMD and cataract were not available in the literature. Instead, these incidence rates were either age- or myopia-specific only, in which case we prefer myopia-specific rates over age-specific rates. This would potentially affect the timing of disease development for individuals, but on average, the lifetime cumulative incidence of severe visual impairment for the simulated population in the model, matched the observed prevalence. One-way sensitivity analyses also showed that varying the incident rates resulted in only small deviations to the ICER and did not affect our conclusions.

## CONCLUSIONS

The use of DIMS to control the progression of myopia during childhood is a cost-effective strategy to reduce the likelihood of ocular complications and poor vision, and to boost QALYs gained over a lifetime. The conclusions hold up well even when the uncertainties around the parameter values are taken into consideration. Further investigation is required to identify cost-effective strategies to address equity in accessing myopia control.

## Additional material


Online Supplementary Document
