# Supplementary material for: Lifetime cost-effectiveness of myopia control intervention for the children population
Source: J Glob Health. 2024 Sep 20;14:04183. doi: 10.7189/jogh.14.04183 (PMC11414424; doi:10.7189/jogh.14.04183)
Supplement: Online Supplementary Document [file jogh-14-04183-s001.pdf]

Figure S1. Simulation flow of retinal detachment. VA – visual acuity

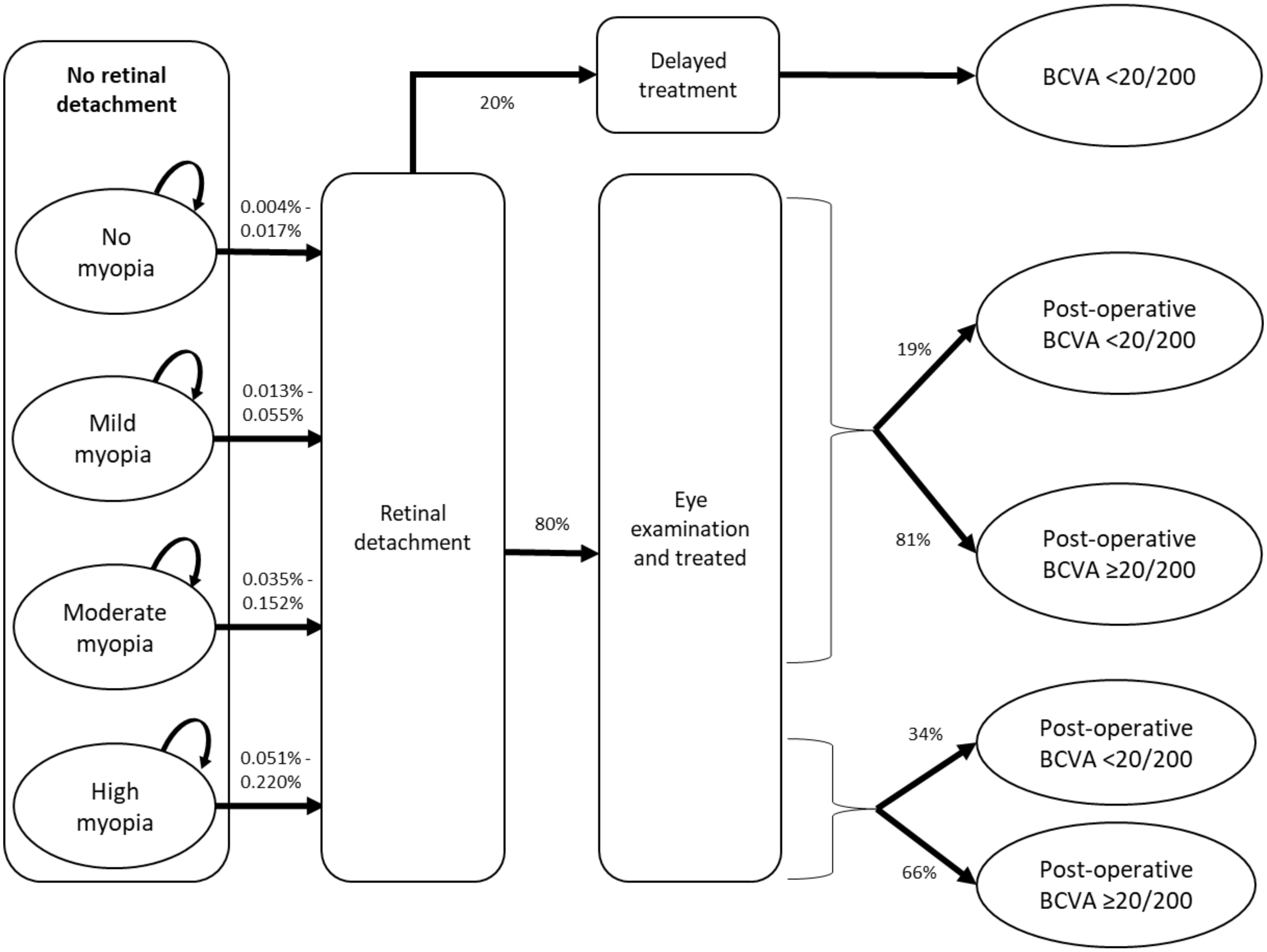

Figure S2. Simulation flow of myopic macular degeneration. META-PM – Meta-Analysis for Pathologic Myopia, MMD – myopic macular degeneration, VA – visual acuity

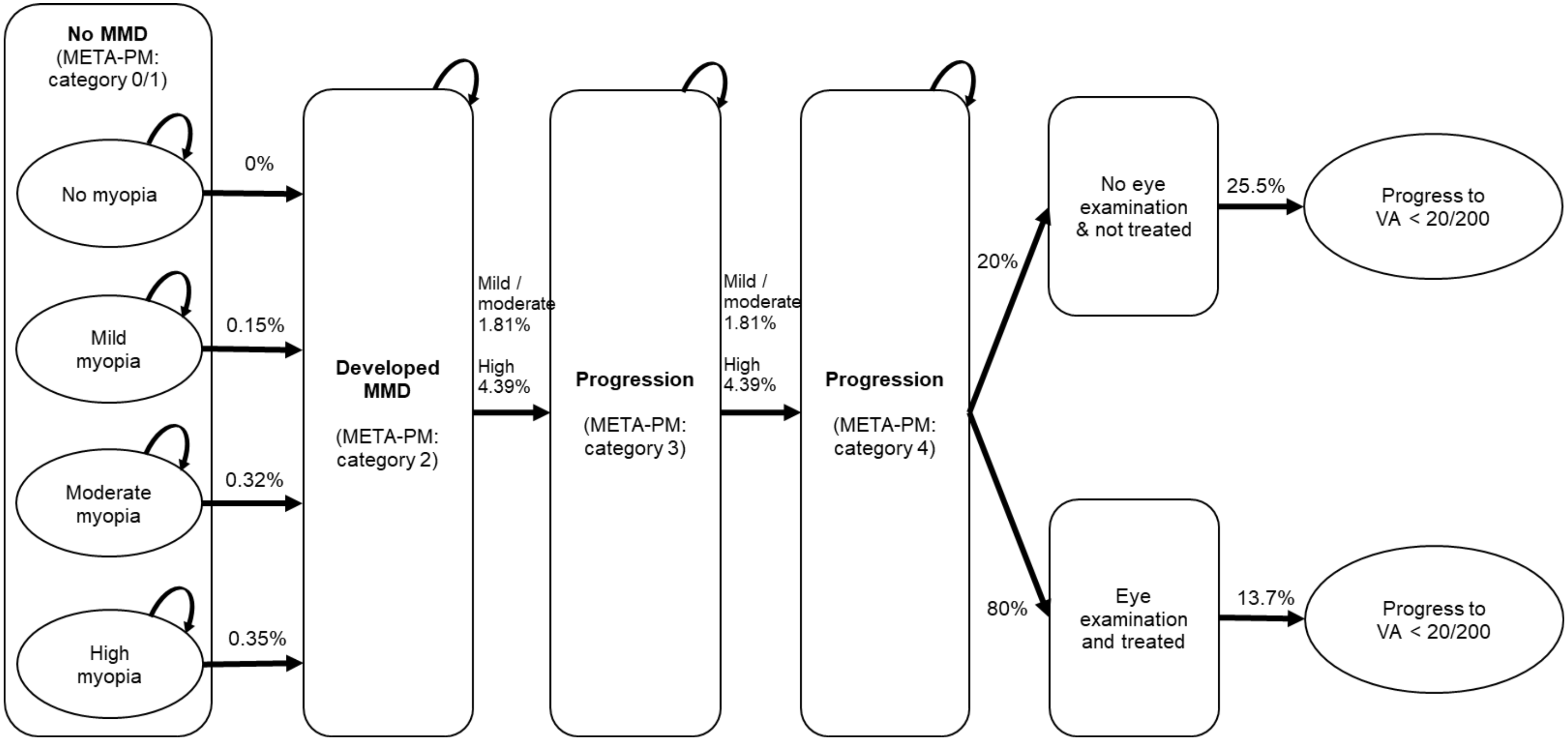

Figure S3. Simulation flow of cataract. CC – cortical cataract, NC – nuclear cataract, PSC – posterior subcapsular cataract

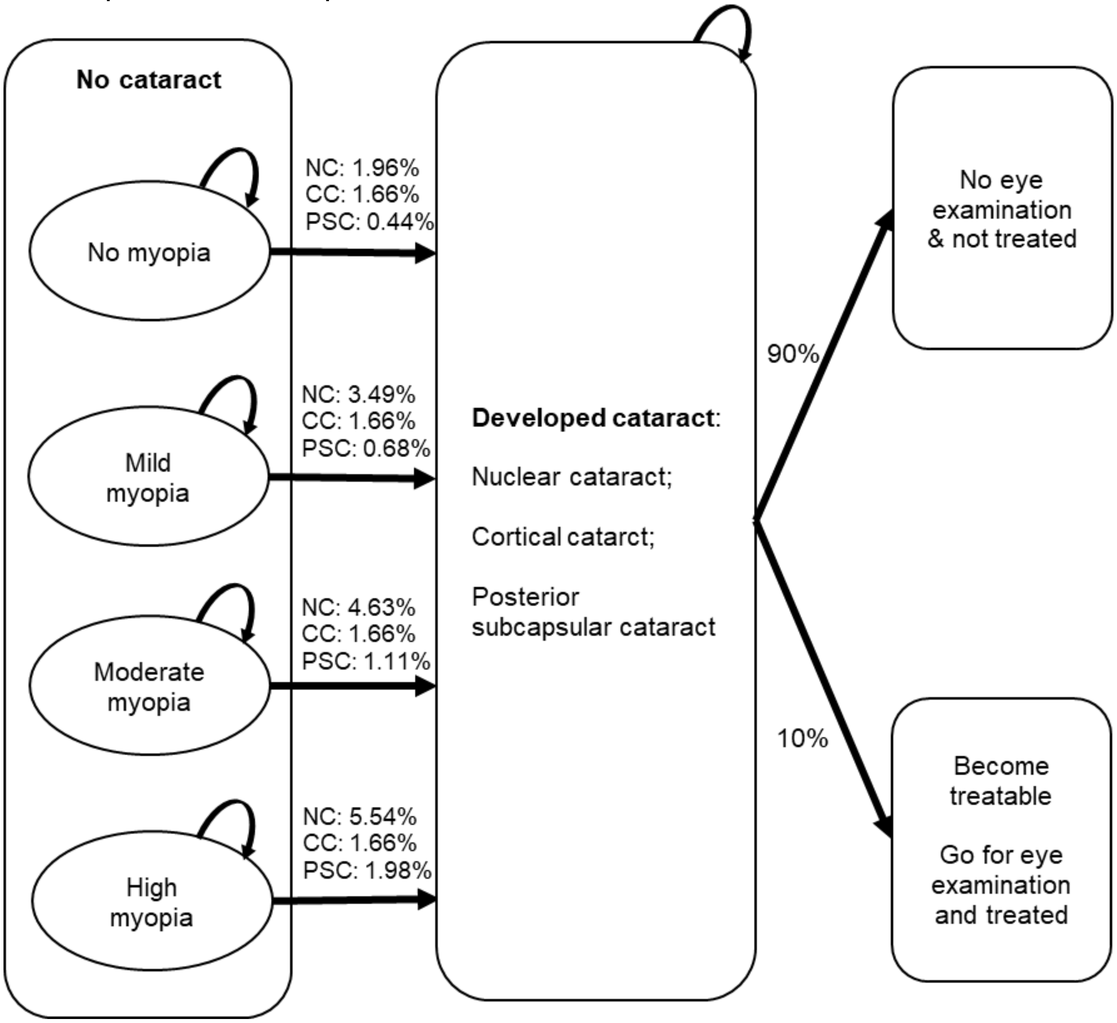

Figure S4. Simulation flow of glaucoma. VA – visual acuity

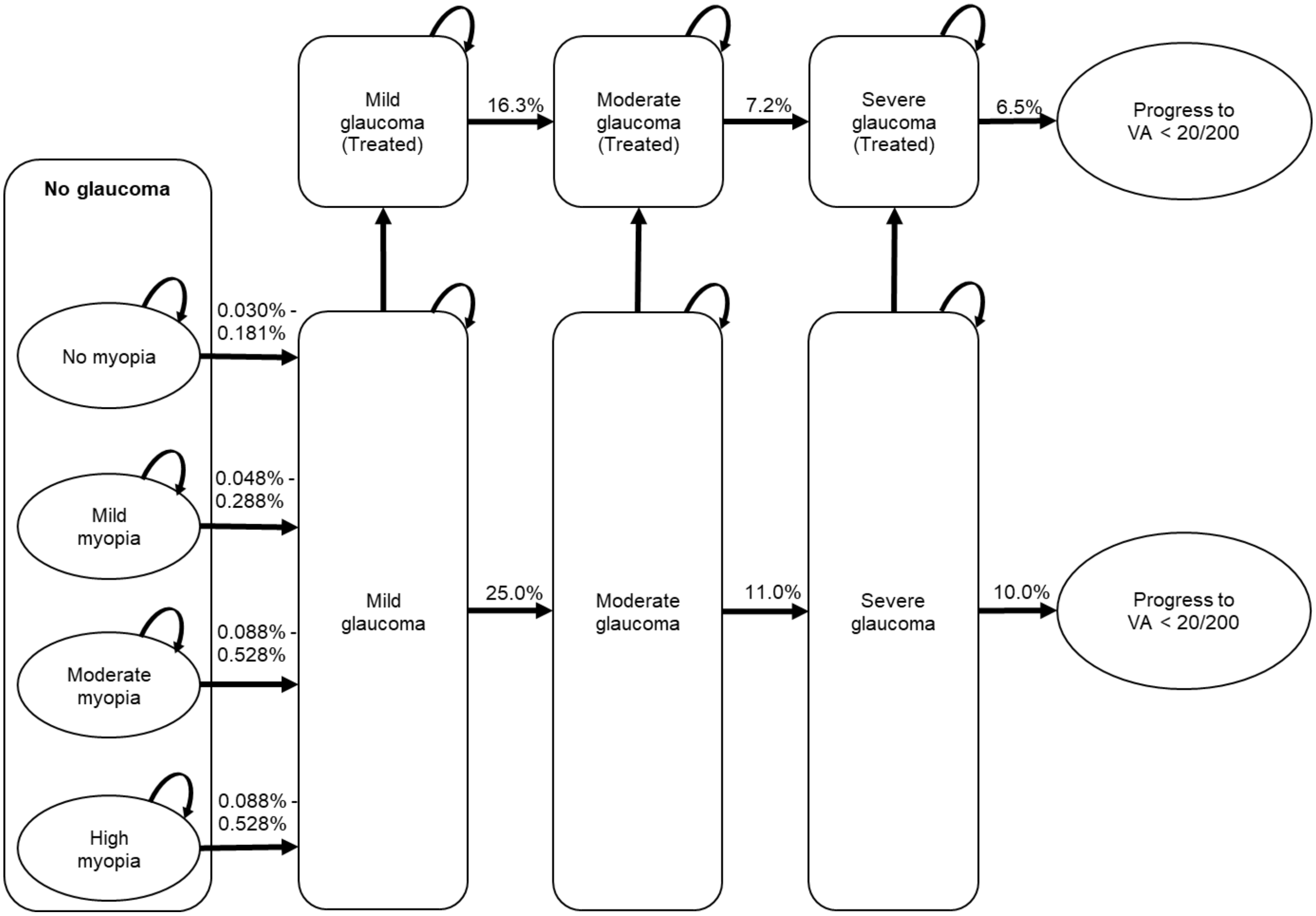

Figure S5. Range and distributions of myopia progression by age and myopia groups.

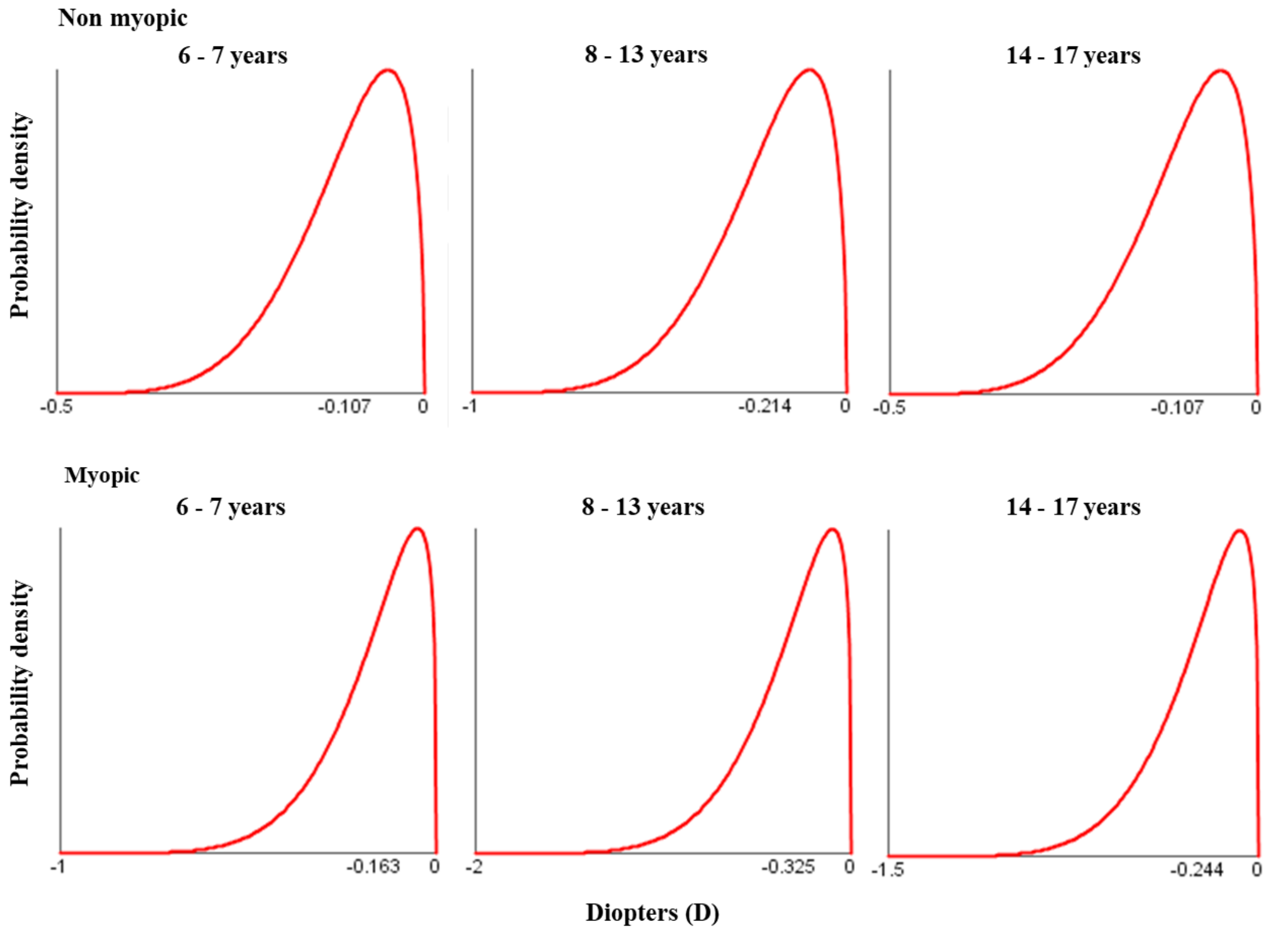

Table S1. Cost-effectiveness results from scenario analyses

	**Relative reduction* in prevalence of high myopia**	**Relative reduction* in severe visual impairment†**	**Incremental cost (USD)**	**QALYs gained**	**Incremental cost per QALYs gained‡ (USD)**
**Base case**					
Discount at 3.5% on costs and QALYs	44.7%	19.2%	1293	0.05	26407
**Scenario analyses**					
Scenario 1 – varying discount rates					
- 3.5% on costs and 0% on QALYs	44.7%	19.2%	1293	0.16	7932
- 3.5% on costs and 5% on QALYs	44.7%	19.2%	1293	0.03	38282
- No discount on costs and QALYs	44.7%	19.2%	-596	0.16	-3654
- 5% on costs and 3.5% on QALYs	44.7%	19.2%	1441	0.05	29419
- 10% on costs and 3.5% on QALYs	44.7%	19.2%	1436	0.05	29321
Scenario 2 – varying uptake of intervention					
- uptake rate at 50%	21.8%	10.4%	642	0.03	22073
- uptake rate at 85%	38.1%	16.7%	1100	0.04	24488
Scenario 3 – simulating a younger children population					
- start with ages 6 – 8	55.4%	20.6%	1332	0.05	25031

QALY – quality-adjusted life year, USD – US dollar
* With myopia control vs. without control
† Visual Acuity < 20/200
‡ May not exactly equal to the costs divided by QALYs, due to rounding of the decimals
